# Supplementary material for: Polymorphism of amyloid-like fibrils can be defined by the concentration of seeds
Source: PeerJ. 2015 Aug 20;3:e1207. doi: 10.7717/peerj.1207 (PMC4563235; doi:10.7717/peerj.1207)
Supplement: Supplemental Information 1 [file peerj-03-1207-s001.pdf]

Supplementary information

Secondary nucleation overcomes seeding template in amyloid-like fibril formation

K. Milto[1,+], T. Sneideris[1,+], V. Smirnovas[1,*]

[1]*Department of Biothermodynamics and Drug Design, Vilnius University Institute of Biotechnology, Vilnius, Lithuania.*

**Correspondence to: vytautas.smirnovas@bti.vu.lt*

[+]*these authors contributed equally to this work*

Fitting

Four main processes (fibril elongation, primary nucleation, secondary nucleation and fibril fragmentation) were used in the description of fibrillar aggregation, similar as described by Meisl et al., 2014.

We assumed the main increase of mass concentration of fibrils $[M]$, comes from elongation and thus is dependent on monomer concentration $[m]$ and fibril end concentration $[P]$:

$$\frac{d[M]}{dt} = k_E[m][P]$$

In case of primary nucleation we assumed nucleus size of 2 and negligible change in $[M]$, thus it can be described as monomer-dependent increase of fibril ends:

$$\frac{d[P]}{dt} = 2k_N[m]^2$$

The same assumptions were done in case of secondary nucleation, it can be described as monomer and fibril mass-dependent increase of fibril ends:

$$\frac{d[P]}{dt} = 2k_2[m]^2[M]$$

Fibril fragmentation can be described as fibril mass-dependent increase of fibril ends:

$$\frac{d[P]}{dt} = k_F[M]$$

All four processes were combined in directed graph (Fig.1) and fitting was performed using rModeler software from Ubicalc (Vilnius, Lithuania, www.ubicalc.eu). All fittings were performed assuming average fibril size equal to 2000 monomers ($[M]/[P]$=1000].

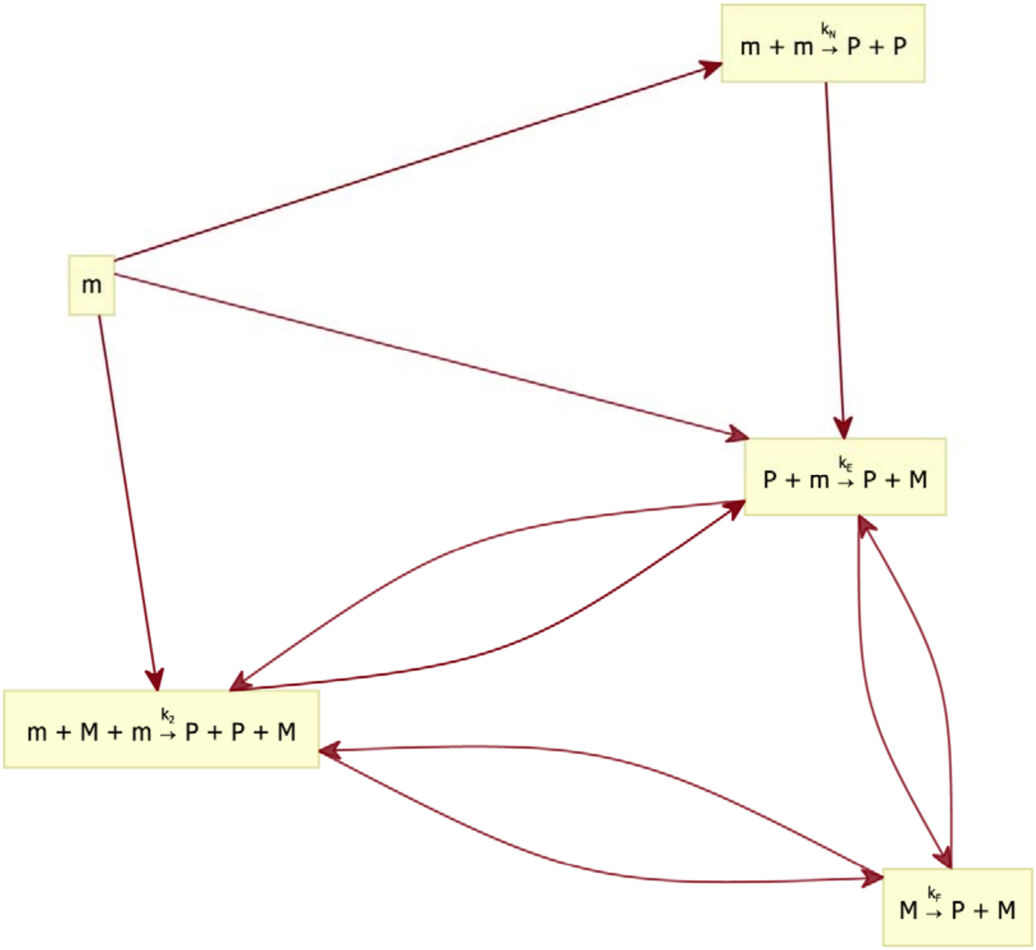

Figure 1. Directed graph of fibrillar aggregation.

Fitting of fibrillation data obtained in presence of 5%, 1% and 0.2% seeds is demonstrated in Figures 2, 3, and 4, respectively. The data obtained in presence of 5% seeds shows good repeatability and the fit of five data repeats result in $k_E \sim 10^2$ $M^{-1}s^{-1}$, while $k_2 < 10^{-10}$ and $k_F < 10^{-10}$. The data obtained in presence of 1% seeds has poor repeatability in general; however the profile shapes are reproducible. It is worth to mention that even a single curve fit is not too good, suggesting the used model to be not sufficient for comprehensive description of the data. The the fit of five data repeats result in $k_E \sim 10^1$ $M^{-1}s^{-1}$, while $k_2 \sim 5 \cdot 10^{-6}$ and $k_F \sim 3 \cdot 10^{-6}$. Finally in case of the data obtained in presence of 0.2% seeds the the fit of five data repeats result in $k_E \sim 4 \cdot 10^{-1}$ $M^{-1}s^{-1}$, while $k_2 \sim 5 \cdot 10^{-4}$ and $k_F \sim 5 \cdot 10^{-4}$. We believe this fitting data gives a hint that the change in the shape of the curve is because of the change from elongation-driven process towards secondary nucleation/fragmentation-driven process.

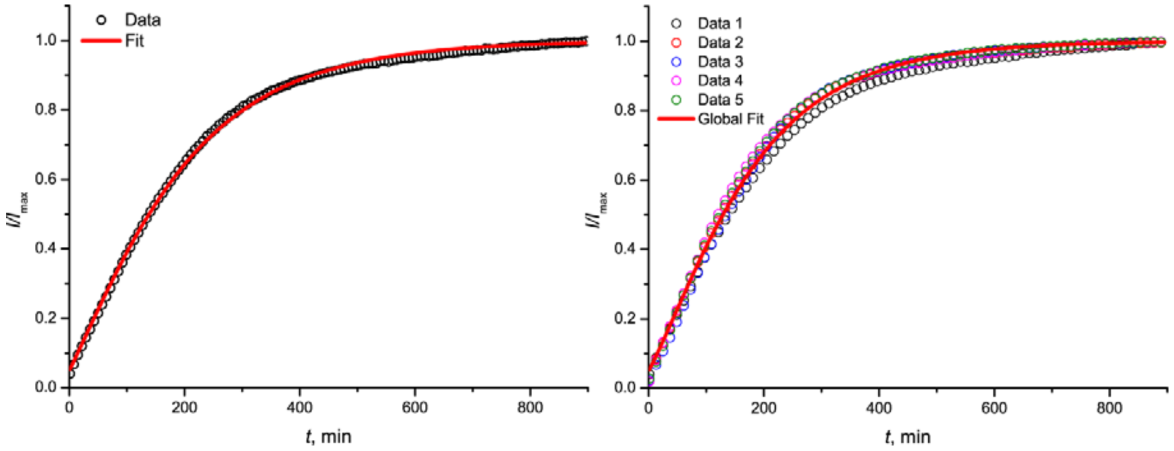

Figure 2. Fitting of fibrillation in presence of 5% seeds.

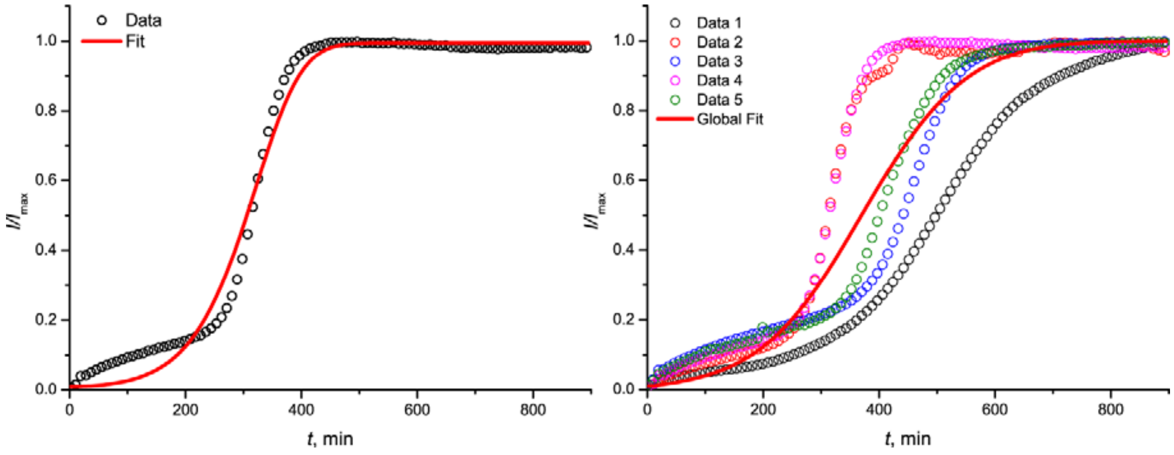

Figure 3. Fitting of fibrillation in presence of 1% seeds.

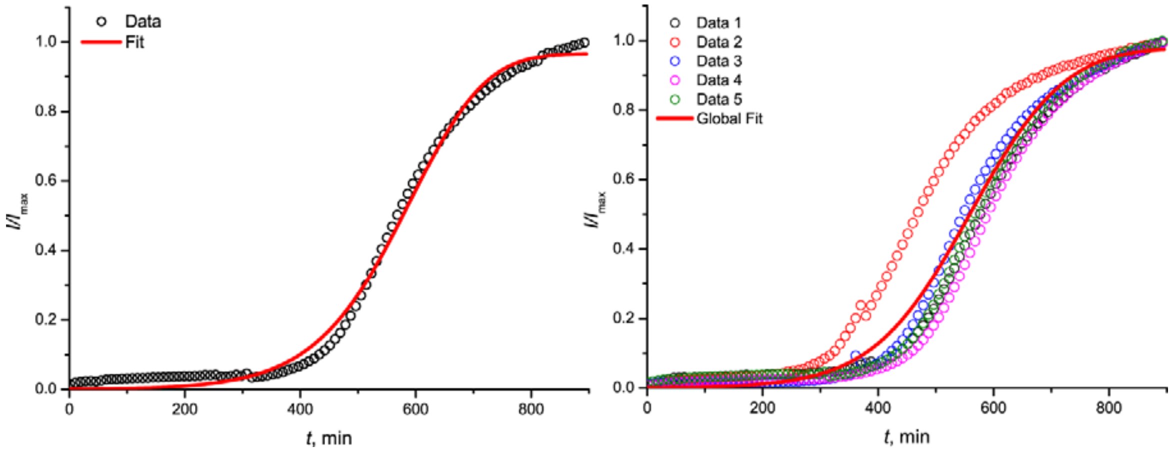

Figure 4. Fitting of fibrillation in presence of 0.2% seeds.
